# The Novel Fusion Protein Melittin‐MIL‐2 Exhibits Strong Antitumor Immune Effect in Lung Adenocarcinoma Cell A549

**DOI:** 10.1111/crj.13805

**Published:** 2024-07-14

**Authors:** Weize Gao, Wenshuai Li, Zhan Wang, Yongxin Li, Mingjun Liu

**Affiliations:** ^1^ Department of Clinical Laboratory, Key Laboratory of Laboratory Medicine The Affiliated Hospital of Qingdao University Qingdao China

**Keywords:** fusion protein, immune effect, lung adenocarcinoma, melittin, rIL‐2

## Abstract

In previous studies, we developed a novel fusion protein named “melittin‐MIL‐2” which exhibited more anti‐tumor activity. However, it remains unclear whether melittin‐MIL‐2 possesses antitumor immune effect on lung adenocarcinoma. In this study, the immune effect and mechanism of melittin‐MIL‐2 inhibiting the growth and invasion of lung adenocarcinoma will be investigated, in order to provide novel perspectives for the immunotherapy of lung cancer. The results indicated that melittin‐MIL‐2 promoted T cell proliferation, enhanced NK cell cytotoxicity, and boosted IFN‐γ secretion in PBMCs. After melittin‐MIL‐2 stimulation, perforin expression and LAK/NK‐like killing activities of human PBMCs and NK cells were significantly enhanced. Melittin‐MIL‐2 is capable of hampering the development and proliferation of lung adenocarcinoma cell A549. ICAM‐1 and Fas expression in A549 cells exposed to melittin‐MIL‐2 rose significantly. The expression levels of TLR8 and VEGF in A549 cells decreased significantly after melittin‐MIL‐2 stimulation. In vivo, melittin‐MIL‐2 substantially impeded the growth of lung adenocarcinoma and formed an immune‐stimulating microenvironment locally in tumor tissues. In conclusion, the novel fusion protein melittin‐MIL‐2 exhibits strong anti‐tumor immune effect in lung adenocarcinoma cell A549 via activating the LFA‐1/ICAM‐1 and Fas/FasL pathways to enhance cytolytic activity, upregulating the secretion of IFN‐γ and perforin, and boosting LAK/NK‐like killing activities. Immuno‐effector cells and their secreted cytokines can form immune stimulation microenvironment locally in lung adenocarcinoma Lewis mice tissue.

## Graphical abstract

The novel fusion protein melittin‐MIL‐2 may activate immune cells via activating the LFA‐1/ICAM‐1 and Fas/FasL pathways to enhance cytolytic activity, upregulating the production of IFN‐γ and perforin, and boosting LAK‐like and NK‐like killing activities. Immuno‐effector cells and their secreted cytokines can form immune stimulation microenvironment locally in lung adenocarcinoma tissue and exert stronger antitumor immune effect.

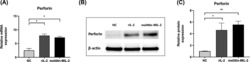

## 1 Introduction

The current treatment methods for lung adenocarcinoma include radiotherapy, chemotherapy, and surgery. However, due to the existence of micrometastases and the fact that a large proportion of individuals are in the middle or late stages when diagnosis, the treatment effect of conventional therapies is not ideal [1, 2, 3]. Tumor immunotherapy, proposed in the early 2000s, brought new hope to lung cancer patients [4, 5, 6, 7]. At present, the research trend of lung cancer immunotherapy is to enhance the anti‐tumor activity of the immune system by activating the immune effector cells, or to attack the tumor by removing the restriction on the immune system to restore its normal function [4, 5, 6, 7]. A variety of immune checkpoint inhibitors, including anti‐CTLA4 monoclonal antibody (mab) ipilimumab, anti‐PD‐1 mab pembrolizumab and nivolumab, and anti‐PD‐L1 mab atezolizumab and durvalumab, have been used for the therapy of non–small cell lung cancer (NSCLC) [8, 9]. Furthermore, cytokine fusion proteins, cancer vaccines, therapeutic antibodies, and cell therapy have been used in lung cancer immunotherapy research [10].

In previous study, we recombined melittin with the mutant interleukin‐2 (IL‐2) to form a novel fusion protein melittin‐MIL‐2 (IL‐2 mutant) [11]. Melittin‐MIL‐2 can enhance the anti‐tumor immune effect of recombinant IL‐2 (rIL‐2) monomer and reduce the toxic side effects of rIL‐2 alone. Melittin‐MIL‐2 induced proliferation of cytotoxic T lymphocyte (CTL) and enhanced natural killer (NK) cytotoxicity, impeded the development and proliferation of ovarian cancer cells SKOV3, liver cancer cells SMMC‐7721, and other tumor cells in vitro. In vivo, melittin‐MIL‐2 impeded cancer development in ovarian and liver cancer mice [11, 12, 13]. Although these findings imply that this recombinant fusion protein has the potential to cure a variety of malignancies, the immunological effect of melittin‐MIL‐2 in lung adenocarcinoma remains unclear. In this study, the immune effect and mechanism of melittin‐MIL‐2 inhibiting the growth and invasion of lung adenocarcinoma will be investigated, in order to provide novel perspectives for the immunotherapy of lung cancer.

## 2 Materials and Methods

### 2.1 Reagents, Cell Lines, and Animals

Sources of melittin‐MIL‐2, rIL‐2, and ELISA kits are described in the previous study [13]. The primers were synthesized by Shanghai Sangon. The primary antibody for Western blot was purchased from Abcam (Shanghai, China). Secondary antibodies were purchased from Proteintech (Wuhan, China). CTLL2 cells were obtained from Shanghai EK‐Bioscience Biotechnology Co., Ltd. (Id:ATCC TIB‐214). K562 cells (Id:SCSP‐5054) and A549 cells (Id:SCSP‐503) were obtained from the Shanghai Cell Bank of the Chinese Academy of Sciences. C57BL/6 mice (6 weeks old) were purchased from the Chinese Academy of Medical Sciences. This study included three healthy patients who received medical examination in the Affiliated Hospital of Qingdao University in September 2020. Collect the remaining blood samples of the subjects after they have completed routine testing. Peripheral blood mononuclear cells were isolated by Ficoll–Hypaque method. All subjects gave their informed consent for inclusion before they participated in the study. The study was conducted in accordance with the Declaration of Helsinki, and the protocol was approved by the Ethics Committee of Affiliated Hospital of Qingdao University (QYFYWZLL27979). The experiment was repeated at least three times. Each experiment consisted of three technical replicates.

### 2.2 Proliferation Assay, Cytotoxicity Assay, and IFN‐γ Release Assay (ELISA)

The detailed protocol of the proliferation assay and cytotoxicity assay was described in the previous study [13]. In this study, human lung cancer A549 was used as target cells.

### 2.3 Perforin Expression and Lymphokine‐Activated Killer (LAK)‐ and NK‐Like Cytotoxicity of Melittin‐MIL‐2‐Activated Human PBMCs

Total RNA was extracted by Trizol method, and then, RT‐PCR was performed. Perforin primer sequence is as follows: forward 5′TGTATGATGGCTGGGG3′; reverse 5′CCTGTGGTAAGCATGCT3′. The β‐actin gene was used as an internal control. β‐Actin primer sequence is as follows: forward 5′ACCTTCTACAATGAGCTGCG 3′; reverse 5′TGCTTGCTGATCCACATCTGC3′. Total protein was extracted from the cells after RIPA lysis, and the perforin was detected by Western blot. A549 and K562 cells in logarithmic growth phase were used as target cells for LAK‐like and NK‐like killing assays, respectively. And the above two target cells were added into 96 well plates. PBMCs were induced by melittin‐MIL‐2 or rIL‐2 for 5 days to become LAK cells. LAK cells obtained by the above methods were used in LAK‐like killing assays as effector cells to attack A549 cells. In the NK‐like killing assay, NK cells in PBMCs were sorted out by immunomagnetic bead sorting technique as described in previous study [13]. The sorted NK cells were then induced with melittin‐MIL‐2 or rIL‐2 for 5 days and then used as effector cells to kill K562 cells. K562 is commonly used as an important tool cell line to detect the expansion and activation of NK cells. The density of effector cells was adjusted according to the effector‐target ratio of 5 : 1, 10 : 1, 20 : 1, and 40 : 1 and then added into the target cell plate. Target cell control and fresh and activated effector cell control at different concentrations were also set up. The cells were incubated for 24 h at 37°C with 5% CO2. The OD value was detected by microplate reader, and the killing rate was calculated according to the following formula: the killing rate (%) = [1 − (OD value of experimental wells − OD value of effector cells control wells)/OD value of target cells control wells] × 100%.

### 2.4 Detection of Intercellular Adhesion Molecule (ICAM‐1), Fas, TLR8, and VEGF on the Surface of A549 Cells and A549 Cell Proliferation Inhibition Assay

Western blot was applied to evaluate the expression of ICAM‐1 (ab282575, Abcam) and Fas (ab133619, Abcam) in human lung adenocarcinoma A549 cells stimulated by melittin‐MIL‐2 or rIL‐2. qRT‐PCR and Western blot were applied to evaluate the expression of TLR8 (ab180610, Abcam), VEGFC (ab9546, Abcam), and VEGFD (ab155288, Abcam) in human lung adenocarcinoma A549 cells stimulated by melittin‐MIL‐2 or rIL‐2. TLR8 primer sequence is as follows: forward 5′CAACCAAAGCAAGAAAACA 3′; reverse 5′TGCAAAGCCAAGTAAAAA 3′. VEGF primer sequence is as follows: forward 5′AATGTGGGGCCAACCGAGAA3′; reverse 5′CCAATATGAAGGGACACAACG3′. MTT assay was used to evaluate the growth inhibitory effect of melittin‐MIL‐2 on lung adenocarcinoma A549 cells. The detailed protocol was described in the previous study.

### 2.5 Lung Adenocarcinoma Growth and Invasion Inhibition Assay

Lewis lung adenocarcinoma model was established by subcutaneous injection of 3.0 × 10^6^ Lewis lung adenocarcinoma cells (LLCs) in C57BL/6 mice. The LLC used in vivo was obtained from the Shanghai Cell Bank of the Chinese Academy of Sciences. A total of 30 C57BL/6 mice were used in vivo. Ten mice were included in each group (PBS, rIL‐2, and melittin‐MIL‐2). The mice were administrated with PBS, melittin‐MIL‐2, and rIL‐2 by intraperitoneal injection (200 M/mouse) on the day of Lewis cell inoculation. After inoculation of LLCs for 7 days, the tumor growth was observed and the tumor volume was calculated every day. On Day 54 after administration, mice were sacrificed by neck amputation under anesthesia, and tumor tissues were removed and the tumor volume was calculated for the last time.

Tumor volume (mm^3^) = [maximum tumor diameter (mm) × shortest tumor diameter (mm)^2^]/2.

### 2.6 Assay of Cytokines Released From Lung Adenocarcinoma Tissue

To evaluate the immune efficacy of melittin‐MIL‐2 against lung adenocarcinoma in mice and to evaluate whether melittin‐MIL‐2 could form an immune‐stimulating microenvironment in lung adenocarcinoma tissues, mice were killed 30 days after inoculation of lung adenocarcinoma cells and collected tumor tissues. The tumor tissues were then homogenized in PBS, and the cytokine concentrations of IFN‐γ, tumor necrosis factor (TNF)‐α, IL‐12, and IL‐4 in each sample were detected by ELISA kits.

### 2.7 Statistical Analysis

Statistical data are presented as mean ± SD. The statistical significance of the differences between the groups was examined using the Student's *T* test. A difference was deemed statistically significant if the *p* value was less than 0.05. The computations were performed using SPSS Version 26.0 for Windows software from SSPS Inc. in Chicago, United States.

## 3 Results

### 3.1 Melittin‐MIL‐2 Exhibits High Biological Activity

After the novel fusion protein melittin‐MIL‐2 has been expressed and purified, its associated biological activity must first be evaluated in order to further investigate its immune effect and mechanism in suppressing the proliferation of lung adenocarcinoma cell A549. In this study, the biological activity of melittin‐MIL‐2 was examined (Figures S1 and S2). The results were similar to our previous study [12, 13].

### 3.2 The Melittin‐MIL‐2 Upregulated the Expression of Perforin and Enhanced the LAK‐Like and NK‐Like Killing Effects of PBMCs

**FIGURE 1:**
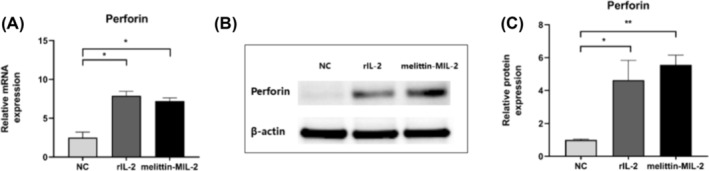
The melittin‐MIL‐2 upregulated perforin expression. (A) Fresh PBMCs and melittin‐MIL‐2 or rIL‐2 activated PBMCs for 5 days were collected. Following the Trizol technique for total RNA extraction, RT‐PCR was carried out. Perforin mRNA was expressed at a significantly higher level in PBMCs that had been stimulated by melittin‐MIL‐2 in contrast with the control group (*p < 0.05). (B, C) The same trend was observed at the protein level. The β‐actin band in (B) is similar with that in Figure 5B. The two target proteins, perforin and TLR8, were detected by Western blot on the same membrane (**p < 0.01).

**FIGURE 2:**
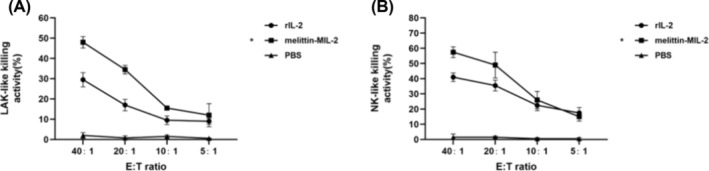
The melittin‐MIL‐2 enhanced the LAK‐like and NK‐like killing effects of PBMCs and NK cells. (A) Lung adenocarcinoma cell A549 at the logarithmic growth stage were planted in 96‐well plates as target cells for LAK‐like killing. PBMCs were induced by melittin‐MIL‐2 or rIL‐2 for 5 days to become lymphokine‐activated killer (LAK) cells. LAK cells obtained by the above methods were used in LAK‐like killing assays as effector cells to attack A549 cells. The density of effector cells was adjusted according to 5 : 1, 10 : 1, 20 : 1, and 40 : 1 E:T ratios and then added into the target cell plate. Compared with rIL‐2 and PBS, the LAK‐like killing activity of PBMCs activated by melittin‐MIL‐2 was significantly enhanced at 40:1 and 20:1 E:T ratios (*p < 0.05). (B) K562 cells at the logarithmic growth stage were planted in 96‐well plates as target cells for NK‐like killing assay. In the NK‐like killing assay, NK cells in PBMCs were sorted out by immunomagnetic bead sorting technique. The sorted NK cells were then induced with melittin‐MIL‐2 or rIL‐2 for 5 days and then used as effector cells to kill K562 cells. The density of effector cells was adjusted according to 5 : 1, 10 : 1, 20 : 1, and 40 : 1 E:T ratios and then added into the target cell plate. The NK‐like killing activity of NK cells activated by melittin‐MIL‐2 was significantly enhanced in contrast with rIL‐2 and PBS at 40:1 E:T ratios (*p < 0.05).

After activation of human PBMCs with melittin‐MIL‐2, the expression level of perforin mRNA was significantly increased, which was considerably higher than that of the control group (Figure 1A). In addition, the protein level of perforin was also significantly increased after PBMCs were stimulated with melittin‐MIL‐2 (Figure 1B,C) Additionally, at 40:1 and 20:1 effector‐to‐target ratios, the LAK‐like killing activity of PBMCs induced by melittin‐MIL‐2 was dramatically increased as compared to rIL‐2 and PBS (Figure 2A). MTT tests also demonstrated that PBMCs activated by melittin‐MIL‐2 had considerably higher NK‐like killing activity at a 40:1 effector‐to‐target ratio than rIL‐2 and PBS (Figure 2B).

### 3.3 The Melittin‐MIL‐2 Inhibited Cell Proliferation and Upregulated the Expression of ICAM‐1 and Fas in A549 Cells

**FIGURE 3:**
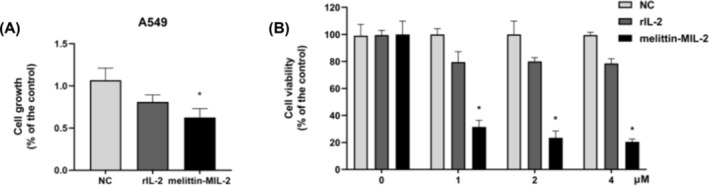
The melittin‐MIL‐2 inhibited A549 cell proliferation. (A) The melittin‐MIL‐2 inhibited cell proliferation (*p < 0.05). (B) The viability of A549 cells in the melittin‐MIL‐2 treatment group dropped considerably in a dose‐dependent manner (*p < 0.05).

**FIGURE 4:**
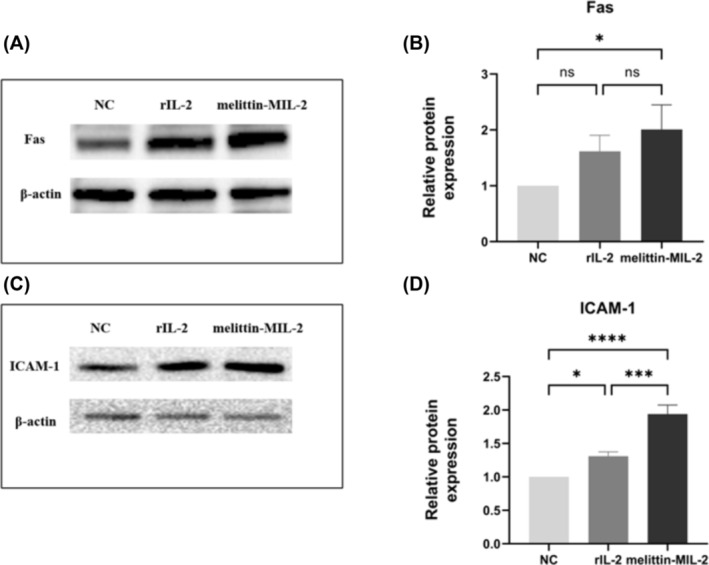
The melittin‐MIL‐2 upregulated the expression of Fas and ICAM‐1 in A549 cells. (A, B) After being exposed to melittin‐MIL‐2 for 5 days, the expression level of Fas in A549 cells was significantly higher than that in the control. However, the expression level of Fas did not reach statistical difference between the rIL‐2 and control groups (*p < 0.05). (C, D) Both rIL‐2 and melittin‐MIL‐2 significantly upregulated the expression of ICAM‐1 in A549 cells, which was statistically different from the control group. More importantly, melittin‐MIL‐2 was more potent than rIL‐2 in stimulating ICAM‐1 expression in A549 cells (*p < 0.05, ***p < 0.001, ****p < 0.0001).

MTT results showed that melittin‐MIL‐2 impeded the development and proliferation of lung adenocarcinoma cell A549 (Figure 3A). In Figure 3A, rIL‐2 also significantly inhibited cell proliferation than control. There were statistical differences between the two groups (*p < 0.05). However, a statistical difference had not yet been reached between the rIL‐2 and melittin‐MIL‐2 groups. To investigate further the concentration‐dependent inhibition of proliferation of lung adenocarcinoma A549 cells, we exposed them to various concentrations of melittin‐MIL‐2, as well as the same diluted concentrations of rIL‐2 as controls. As demonstrated in Figure 3B, the viability of A549 cells in the melittin‐MIL‐2 and rIL‐2 treatment groups dropped considerably with increasing concentration, in a dose‐dependent manner. The optimal melittin‐MIL‐2 concentration was 4 μM, which resulted in 74% growth inhibition. After being exposed to melittin‐MIL‐2 for 5 days, the expression level of Fas in A549 cells was significantly higher than that in the control. However, the expression level of Fas did not reach statistical difference between the rIL‐2 and control groups (Figure 4A,B). Both rIL‐2 and melittin‐MIL‐2 significantly upregulated the expression of ICAM‐1 in A549 cells, which was statistically different from the control group. More importantly, melittin‐MIL‐2 was more potent than rIL‐2 in stimulating ICAM‐1 expression in A549 cells (Figure 4C,D).

### 3.4 The Melittin‐MIL‐2 Downregulated the Expression Levels of TLR8 and VEGF in A549 Cells

**FIGURE 5:**
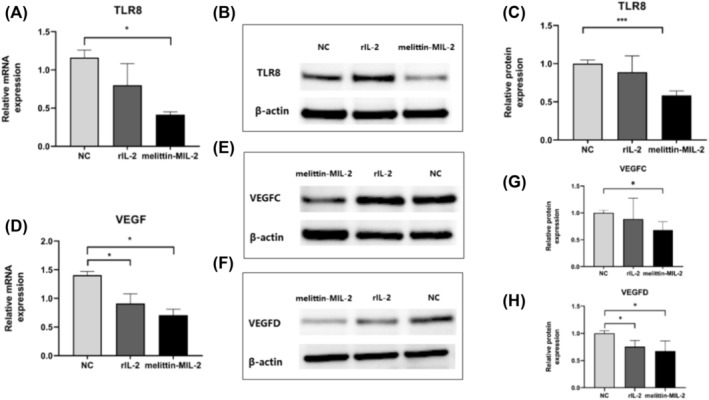
The melittin‐MIL‐2 downregulated the expression levels of TLR and VEGF in A549 cells. (A–C) TLR8 mRNA and protein expression levels in human lung adenocarcinoma cell A549 considerably reduced when compared to rIL‐2 and control. The β‐actin band in (B) is similar with that in Figure 1B. The two target proteins, TLR8 and perforin, were detected by Western blot on the same membrane. (D–H) When compared to the rIL‐2 and control groups, VEGF mRNA and protein expression levels were lower in the melittin‐MIL‐2 treatment group.

Data analysis revealed that after stimulation with melittin‐MIL‐2, TLR8 mRNA and protein expression levels in cell A549 considerably reduced when compared to control (Figure 5A–C). Additionally, mRNA and protein expression levels of VEGFC and VEGFD declined in the melittin‐MIL‐2 treatment group in contrast with the rIL‐2 and control groups (Figure 5D–H).

### 3.5 Melittin‐MIL‐2 Impeded Tumor Development and Displayed Stronger Antitumor Properties Than rIL‐2

**FIGURE 6:**
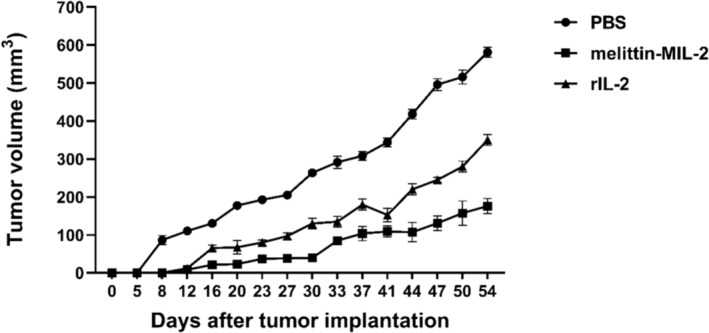
Melittin‐MIL‐2 impeded tumor development and displayed stronger antitumor properties than rIL‐2. A Lewis lung adenocarcinoma mouse model was established by subcutaneous injection of 3.0 × 10^6^ Lewis lung adenocarcinoma cells per cell in pure C57BL/6 mice. PBS, melittin‐MIL‐2, and rIL‐2 were administered by intraperitoneal injection at 200 μM/piece. The average tumor volume among all groups differed significantly after 12 days (*p <* 0.05). From Day 12 through the end of the experiment, the tumor volume in the melittin‐MIL‐2 and control groups differed in a statistically significant way (*p < 0.05). From the 23rd day to the end of the experiment, the tumor volume difference between the melittin‐MIL‐2 and rIL‐2 groups was statistically significant (*p < 0.05).

The capacity of melittin‐MIL‐2 to suppress the development of lung adenocarcinoma A549 cells in vitro suggests that melittin‐MIL‐2 can also exert antitumor activity in vivo. The average tumor volume among all groups differed significantly after 12 days. From Day 12 through the end of the experiment, the tumor volume in the melittin‐MIL‐2 and control groups differed in a statistically significant way. From the 23rd day to the end of the experiment, the tumor volume difference between the melittin‐MIL‐2 and rIL‐2 groups was statistically significant (Figure 6).

### 3.6 Melittin‐MIL‐2 Treatment Formed a Local Immune‐Stimulating Microenvironment

**FIGURE 7:**
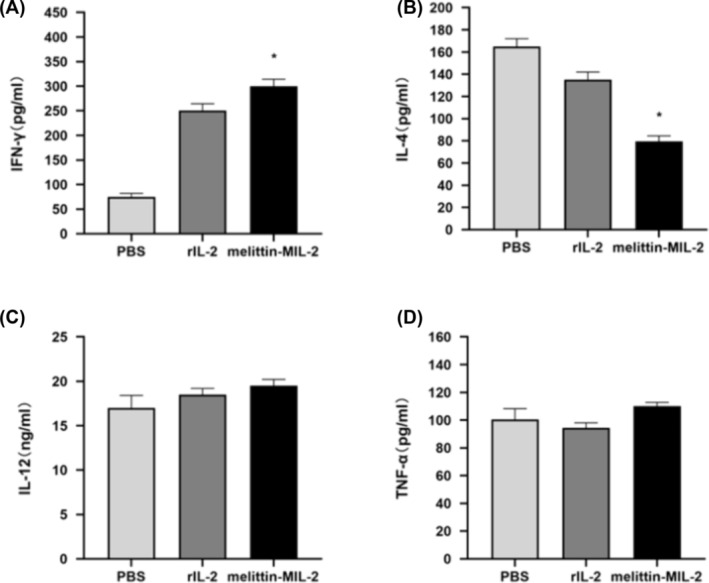
A local immunostimulatory context was created by the melittin‐MIL‐2 therapy. (A) Mice which received melittin‐MIL‐2 treatment had a considerably higher level of IFN‐γ (*p < 0.05). (B) Comparing the fusion protein treated mice to the PBS group, the level of IL‐4 was considerably lower (*p < 0.05). (C, D) There was no discernible change in the levels of IL‐12 and TNF‐α.

For the purpose of determining the immune efficacy of melittin‐MIL‐2 and whether it can form immune‐stimulating microenvironment locally in lung adenocarcinoma tissue, ELISA was used to detect cytokine levels in lung adenocarcinoma tissue. The level of IFN‐γ in the melittin‐MIL‐2 treatment group turned out considerably increased in contrast with the PBS group (Figure 7A). Although IL‐4 levels were significantly decreased in melittin‐MIL‐2‐treated mice, there existed little distinction between the PBS and rIL‐2 groups or between the melittin‐MIL‐2 and rIL‐2 groups (Figure 7B). The results indicated no discernible difference between the PBS‐treated and other groups when IL‐12 was examined (Figure 7C). Furthermore, it showed little difference in TNF‐α levels following intervention using melittin‐MIL‐2 and PBS (Figure 7D).

## 4 Discussion

IL‐2 is secreted by activated T cells in the body, by inducing T cell proliferation differentiation, enhancing cell toxicity of CTL, and promoting NK cells and LAK cells to kill tumor cells [14, 15, 16, 17, 18]. In clinical practice, rIL‐2 has been used for the treatment of malignant tumors such as renal cell carcinoma, melanoma, and liver cancer [19, 20, 21, 22]. However, rIL‐2 demonstrated some drawbacks during therapy, including nonspecific antitumor activity and severe toxicity when utilized in high dosages. The primary explanation for this is that IL‐2 is not very selective for its target receptor. After binding to moderate affinity receptors on the cell surface, IL‐2 may trigger overactivation of NK cells, which leads to excessive secretion of TNF and systemic toxicity [23, 24, 25, 26].

Previous studies have shown that several recombinant human IL‐2 mutant (MIL‐2) can promote antitumor activity while reducing toxicity [20, 25, 27]. Therefore, in our previous study, we constructed a novel IL‐2 mutant MIL‐2 (Arg88/Ala125) [11]. Based on this, we carried out gene chimeric recombination between 26 peptide melittin and IL‐2 mutant MIL‐2 (Arg88/Ala125) before moving on to prokaryotic expression and preliminary eukaryotic expression and purification. The expression product was given the name “melittin‐MIL‐2.” Our prior investigations have shown that the novel cytokine fusion protein “melittin‐MIL‐2” can exert more intense immune effects than IL‐2, inhibit the proliferation of ovarian cancer cell SKOV3 in vitro, and hamper the development of ovarian cancer, liver cancer, and breast cancer metastasis in vivo [12, 13]. These results suggest the promising application of bifunctional molecules composed of toxins and cytokines in the immunotherapy of various tumors.

In the current study, melittin‐MIL‐2 and rIL‐2 demonstrated comparable ability to stimulate the proliferation of activated lymphocytes, proving that melittin‐MIL‐2 still preserved specific IL‐2 activity. Melittin‐MIL‐2 considerably increased the cytolytic activity of human PBMCs against target cells. NK cells treated with melittin‐MIL‐2 had the most notable increase in cytolytic activity. The results could partially be explained by elevated IL‐2 receptor expression on NK cells. Additionally, IFN‐γ secretion was elevated in all melittin‐MIL‐2‐treated NK cells and CD4^+^T and CD8^+^T cells. This is in line with earlier research that found a number of cytokines, including IL‐2, were efficient inducers of IFN‐γ for NK cells and T cells [28, 29, 30, 31]. IFN‐γ is a pleiotropic cytokine, and its antitumor effect varies with the heterogeneity of cancer. IFN‐γ may lead to tumor cell apoptosis directly or indirectly by upregulating the expression of FAS and DR5. IFN‐γ can also exert its killing effect indirectly by making tumor cells susceptible to apoptosis‐inducing immune responses or chemotherapy. Activation of the IFN‐γ/STAT1 pathway can also suppress tumor cell proliferation and induce tumor dormancy [30, 31, 32, 33, 34]. The tumor‐killing action of IFN‐γ in vivo may be directly on tumor cells or indirectly by activation of multiple effect pathways. These include activating macrophages, enhancing T and NK cell function, and promoting MHC antigen expression [28, 30, 31]. Here, the stronger antitumor efficacy of melittin‐MIL‐2 was partially explained by the enhanced IFN‐γ secretion of PBMCs following melittin‐MIL‐2 exposure.

Along with increasing cytolytic activity and IFN‐γ generation, melittin‐MIL‐2 also upregulated the expression of perforin, a mediator associated with PBMCs killing, and enhanced the LAK‐like and NK‐like killing activities of PBMCs and NK cells. When we exposed PBMC target A549 cells to melittin‐MIL‐2, levels of ICAM‐1 and Fas in A549 cells were upregulated. Lymphocyte function‐associated antigen (LFA‐1) is widely present in hematopoietic cells. The ICAM‐1 is a common ligand for LFA‐1 [35, 36]. LFA‐1 is an important promoter of immune synapses formed between CTL cells or NK cells and tumor cells, which mediates the solid adhesion of cytotoxic particles to target cells and directions of adhesion [36, 37]. ICAM‐1 acts as the major ligand of LFA‐1, which may be helpful in immunosurveillance processes [35, 38, 39, 40, 41, 42, 43, 44]. According to these findings, melittin‐MIL‐2 may activate immune cells via activating the LFA‐1/ICAM‐1 and Fas/FasL pathways to enhance cytolytic activity, upregulate the secretion of IFN‐γ and perforin, and boost LAK‐like and NK‐like killing activities.

In this work, lung adenocarcinoma cell A549 proliferation was considerably and dose dependently suppressed by melittin‐MIL‐2. rIL‐2, on the other hand, did not exhibit a comparable inhibitory impact on cell growth. These experimental results provided additional plausible support for the hypothesis that recombinant proteins of melittin and IL‐2 can exert more effective antitumor effects than IL‐2 monomers. TLR signaling pathway might contribute to the formation and growth of cancers [45, 46, 47]. TLR8, as members of the TLR family, are highly expressed in lung cancer tissues and play a role in chronic inflammation, tumor formation, and metastasis. In vitro, TLR8 agonist stimulation of lung cancer cells resulted in the activation of NF‐κB, increased expression of proinflammatory factors (IL‐6, IL‐8, GM‐CSF, IL‐1α, and IL‐12), and increased expression of antiapoptotic proteins Bcl‐2, VEGFR2, and chemokine receptors [48]. Here, levels of TLR‐8 and VEGF were declined in A549 lung adenocarcinoma cells exposed to melittin‐MIL‐2 in vitro. Therefore, we hypothesized that melittin‐MIL‐2 suppressed the proliferation of human lung adenocarcinoma cell A549 by downregulating the expression of TLR8 and VEGF, thereby playing a role in inducing cell apoptosis.

Lewis cells are mouse‐derived lung adenocarcinoma cells [49]. It is a stable cell line isolated from mouse lung adenocarcinoma tissues [49]. Lewis mice had stable growth in tumor volume, showed necrosis later, and had the highest rate of lung metastasis [50]. It is more suitable for experiments of longer duration [50]. Therefore, in order to observe the effect of melittin‐MIL‐2 in inhibiting tumor growth in vivo for 54 days, Lewis lung adenocarcinoma model was established and used in our study. Melittin‐MIL‐2 restrained the tumor development of lung adenocarcinoma more effectively than rIL‐2 in vivo. The tumor microenvironment is an intricate and comprehensive system, which is composed of a variety of stromal cells, including immune cells, adipocytes, fibroblast stromal cells, smooth muscle cells, and the cytokines that play a role in the interaction between tumor cells and the microenvironment [51, 52, 53, 54, 55]. The tumor microenvironment is crucial in deciding whether the tumor gets eradicated or spreads. The ratio of immunosuppressive to immunostimulatory cells and molecules, such as cytokines, creates a bidirectional network in diseased states that can either suppress or advance tumor tissues [52, 53, 54, 55, 56]. IFN‐γ levels in the lung adenocarcinoma tissues of mice significantly rose following melittin‐MIL‐2 injection therapy. IL‐4 levels were significantly reduced, while IL‐12 and TNF‐α levels did not change significantly. The increase of IFN‐γ in the tumor site indicates the presence of CTL and NK cells in the tumor tissue. This could trigger the existence of MHC molecules on the surface of tumor cells, leading to elevated cell death, which is rooted in the provision of tumor immunogenicity [57]. Moreover, a number of studies have shown that a higher number of CTL infiltrations in tumor tissues are associated with a better prognosis for patients. Th cells, a heterogeneous group of immune cells, are also present in the tumor microenvironment. Th1 cells secrete IL‐2 and IFN‐γ to promote cellular immune response, while Th2 cells inhibit antitumor cell immune response by secreting IL‐4, IL‐5, IL‐10, IL‐13, and other cytokines [58, 59]. Melittin‐MIL‐2 injection upregulated IFN‐γ levels as well as inhibited IL‐4 secretion, facilitating the development of Th1 subtypes relative to Th2 cells in lung adenocarcinoma microenvironment. These results confirmed that melittin‐MIL‐2 can form a local immune‐stimulating microenvironment in vivo, which is conducive to reducing immune‐suppression and promoting tumor clearance.

There are several shortcomings in this study. Firstly, this study focused on lung adenocarcinoma A549 cells to investigate the antitumor effects and mechanisms of melittin‐MIL‐2. Animal experiments were used only to investigate the effect of melittin‐MIL‐2 on tumor growth inhibition. Secondly, given the multiple roles of TLR8 and VEGF in vivo, we will measure apoptosis directly in future studies instead of using TLR‐8 and VEGF as surrogates. Finally, we considered the increase in IFN‐γ levels in tumor tissues as changes in CTL and NK cells, but experimental results directly reflecting immune cells were lacking. In future studies, techniques such as flow cytometry or immunohistochemistry will be employed to demonstrate changes in immune cells.

The majority of lung cancers are discovered at an advanced stage, making it one of the most prevalent malignant tumors in clinical practice [60, 61, 62]. The conventional therapy is limited by multiple bottlenecks, and the long‐term effective rate is low, so the patients who undergo early surgery die of recurrence and distant metastasis [63, 64]. As technology has advanced, lung cancer immunotherapy has made strides and is now a viable therapeutic option. In this work, we investigated the immunological mechanism of melittin‐MIL‐2 in lung cancer, providing a fresh perspective on lung adenocarcinoma immunotherapy.

## 5 Conclusions

The novel fusion protein melittin‐MIL‐2 may activate immune cells via activating the LFA‐1/ICAM‐1 and Fas/FasL pathways to enhance cytolytic activity, upregulating the production of IFN‐γ and perforin, and boosting LAK‐like and NK‐like killing activities. The melittin‐MIL‐2 impeded the development and proliferation of lung adenocarcinoma cell A549. Immuno‐effector cells and their secreted cytokines can form immune stimulation microenvironment locally in lung adenocarcinoma tissue and exert strong antitumor immune effect.

## Author Contributions

Weize Gao and Wenshuai Li contributed equally to this work. Weize Gao and Wenshuai Li completed the experiment and wrote the article. Zhan Wang and Yongxin Li contributed significantly to revise and manuscript preparation. Mingjun Liu guided the whole process of this work with constructive discussions.

## Ethics Statement

The study was conducted in accordance with the Declaration of Helsinki, and the protocol was approved by the Ethics Committee of Affiliated Hospital of Qingdao University (QYFYWZLL27979).

## Consent

All subjects gave their informed consent for inclusion before they participated in the study.

## Conflicts of Interest

The authors declare no conflicts of interest.

## Supporting information


**Data S1** Supporting Information

## Data Availability

The data that support the findings of this study are available from the corresponding author upon reasonable request.

## References

[1] E. Kuhn , P. Morbini , A. Cancellieri , S. Damiani , A. Cavazza , and C. E. Comin , “Adenocarcinoma Classification: Patterns and Prognosis,” Pathologica 110, no. 1 (2018): 5–11. 30259909

[2] X. Yin , Y. Li , H. Wang , et al., “Small Cell Lung Cancer Transformation: From Pathogenesis to Treatment,” Seminars in Cancer Biology 86, no. Pt 2 (2022): 595–606, 10.1016/j.semcancer.2022.03.006. 35276343

[3] A. Anichini , V. E. Perotti , F. Sgambelluri , and R. Mortarini , “Immune Escape Mechanisms in Non Small Cell Lung Cancer,” Cancers (Basel) 12, no. 12 (2020): 3605, 10.3390/cancers12123605. 33276569 PMC7761620

[4] Breakthrough of the Year 2013, “Notable Developments,” Science 342, no. 6165 (2013): 1435–1441, 10.1126/science.342.6165.1444. 24357296

[5] M. Aldarouish and C. Wang , “Trends and Advances in Tumor Immunology and Lung Cancer Immunotherapy,” Journal of Experimental & Clinical Cancer Research 35 (2016): 157. 27686848 10.1186/s13046-016-0439-3 PMC5043622

[6] M. Reck , J. Remon , and M. D. Hellmann , “First‐Line Immunotherapy for Non‐Small‐Cell Lung Cancer,” Journal of Clinical Oncology 40, no. 6 (2022): 586–597, 10.1200/JCO.21.01497. 34985920

[7] B. Zulfiqar , A. Farooq , S. Kanwal , and K. Asghar , “Immunotherapy and Targeted Therapy for Lung Cancer: Current Status and Future Perspectives,” Frontiers in Pharmacology 13 (2022): 1035171, 10.3389/fphar.2022.1035171. 36518665 PMC9742438

[8] A. Sgambato , F. Casaluce , P. C. Sacco , et al., “Anti PD‐1 and PDL‐1 Immunotherapy in the Treatment of Advanced Non‐ Small Cell Lung Cancer (NSCLC): A Review on Toxicity Profile and Its Management,” Current Drug Safety 11, no. 1 (2016): 62–68. 26412670 10.2174/1574886311207040289

[9] D. Kazandjian , D. L. Suzman , G. Blumenthal , et al., “FDA Approval Summary: Nivolumab for the Treatment of Metastatic Non‐Small Cell Lung Cancer With Progression on or After Platinum‐Based Chemotherapy,” The Oncologist 21, no. 5 (2016): 634–642, 10.1634/theoncologist.2015-0507. 26984449 PMC4861371

[10] M. Miller and N. Hanna , “Advances in Systemic Therapy for Non‐Small Cell Lung Cancer,” BMJ 375 (2021): n2363, 10.1136/bmj.n2363. 34753715

[11] M. Liu , B. Wang , G. Sun , et al., “Expression, Purification, and Characterization of a Functional Mutant Recombinant Human Interleukin‐2,” Protein and Peptide Letters 17, no. 10 (2010): 1280–1284. 20518734 10.2174/092986610792231474

[12] M. Liu , J. Zong , Z. Liu , et al., “A Novel Melittin‐MhIL‐2 Fusion Protein Inhibits the Growth of Human Ovarian Cancer SKOV3 Cells In Vitro and In Vivo Tumor Growth,” Cancer Immunology, Immunotherapy 62, no. 5 (2013): 889–895, 10.1007/s00262-013-1401-2. 23443963 PMC11029713

[13] M. Liu , H. Wang , L. Liu , B. Wang , and G. Sun , “Melittin‐MIL‐2 Fusion Protein as a Candidate for Cancer Immunotherapy,” Journal of Translational Medicine 14, no. 1 (2016): 155, 10.1186/s12967-016-0910-0. 27246873 PMC4888606

[14] R. Setoguchi , S. Hori , T. Takahashi , and S. Sakaguchi , “Homeostatic Maintenance of Natural Foxp3(+) CD25(+) CD4(+) Regulatory T Cells by Interleukin (IL)‐2 and Induction of Autoimmune Disease by IL‐2 Neutralization,” The Journal of Experimental Medicine 201, no. 5 (2005): 723–735. 15753206 10.1084/jem.20041982 PMC2212841

[15] S. L. Gaffen and K. D. Liu , “Overview of Interleukin‐2 Function, Production and Clinical Applications,” Cytokine 28, no. 3 (2004): 109–123, 10.1016/j.cyto.2004.06.010. 15473953

[16] T. Sato , H. Matsui , S. Shibahara , et al., “New Approaches for the High‐Level Expression of Human Interleukin‐2 cDNA in *Escherichia coli* ,” Journal of Biochemistry 101, no. 2 (1987): 525–534, 10.1093/oxfordjournals.jbchem.a121940. 3294818

[17] L. J. Seigel , M. E. Harper , F. Wong‐Staal , R. C. Gallo , W. G. Nash , and S. J. O'Brien , “Gene for T‐Cell Growth Factor: Location on Human Chromosome 4q and Feline Chromosome B1,” Science 223, no. 4632 (1984): 175–178. 6318318 10.1126/science.6318318

[18] A. K. Abbas , E. Trotta , D. R. Simeonov , A. Marson , and J. A. Bluestone , “Revisiting IL‐2: Biology and Therapeutic Prospects,” Science Immunology 3, no. 25 (2018): eaat1482, 10.1126/sciimmunol.aat1482. 29980618

[19] M. E. Raeber , D. Sahin , and O. Boyman , “Interleukin‐2‐Based Therapies in Cancer,” Science Translational Medicine 14, no. 670 (2022): eabo5409, 10.1126/scitranslmed.abo5409. 36350987

[20] R. Hernandez , J. Põder , K. M. LaPorte , and T. R. Malek , “Engineering IL‐2 for Immunotherapy of Autoimmunity and Cancer,” Nature Reviews. Immunology 22, no. 10 (2022): 614–628, 10.1038/s41577-022-00680-w. 35217787

[21] W. W. Overwijk , M. A. Tagliaferri , and J. Zalevsky , “Engineering IL‐2 to Give New Life to T Cell Immunotherapy,” Annual Review of Medicine 72 (2021): 281–311, 10.1146/annurev-med-073118-011031. 33158368

[22] J. G. Pol , P. Caudana , J. Paillet , E. Piaggio , and G. Kroemer , “Effects of Interleukin‐2 in Immunostimulation and Immunosuppression,” The Journal of Experimental Medicine 217, no. 1 (2020): 1–23, 10.1084/jem.20191247. 31611250 PMC7037245

[23] R. A. Maas , H. F. Dullens , and W. Den Otter , “Interleukin‐2 in Cancer Treatment: Disappointing or (Still) Promising? A Review,” Cancer Immunology, Immunotherapy 36, no. 3 (1993): 141–148. 8439974 10.1007/BF01741084 PMC11038683

[24] U. Dafni , O. Michielin , S. M. Lluesma , et al., “Efficacy of Adoptive Therapy With Tumor‐Infiltrating Lymphocytes and Recombinant Interleukin‐2 in Advanced Cutaneous Melanoma: A Systematic Review and Meta‐Analysis,” Annals of Oncology 30, no. 12 (2019): 1902–1913, 10.1093/annonc/mdz398. 31566658

[25] A. B. Shanafelt , Y. Lin , M. C. Shanafelt , et al., “A T‐Cell‐Selective Interleukin 2 Mutein Exhibits Potent Antitumor Activity and Is Well Tolerated In Vivo,” Nature Biotechnology 18, no. 11 (2000): 1197–1202. 10.1038/81199 11062441

[26] J. Damoiseaux , “The IL‐2 ‐ IL‐2 Receptor Pathway in Health and Disease: The Role of the Soluble IL‐2 Receptor,” Clinical Immunology 218 (2020): 108515, 10.1016/j.clim.2020.108515. 32619646

[27] M. Mizui , “Natural and Modified IL‐2 for the Treatment of Cancer and Autoimmune Diseases,” Clinical Immunology 206 (2019): 63–70, 10.1016/j.clim.2018.11.002. 30415086

[28] F. R. Balkwill , “Interferons,” Lancet 1, no. 8646 (1989): 1060–1063. 2469921 10.1016/s0140-6736(89)92455-0

[29] T. R. Malek , “The Biology of Interleukin‐2,” Annual Review of Immunology 26 (2008): 453–479. 10.1146/annurev.immunol.26.021607.090357 18062768

[30] D. Jorgovanovic , M. Song , L. Wang , and Y. Zhang , “Roles of IFN‐γ in Tumor Progression and Regression: A Review,” Biomarker Research 8 (2020): 49, 10.1186/s40364-020-00228-x. 33005420 PMC7526126

[31] J. D. Burke and H. A. Young , “IFN‐γ: A Cytokine at the Right Time, Is in the Right Place,” Seminars in Immunology 43 (2019): 101280, 10.1016/j.smim.2019.05.002. 31221552 PMC7367502

[32] H. F. Aqbi , M. Wallace , S. Sappal , K. K. Payne , and M. H. Manjili , “IFN‐γ Orchestrates Tumor Elimination, Tumor Dormancy, Tumor Escape, and Progression,” Journal of Leukocyte Biology 103 (2018): 1219–1223, 10.1002/JLB.5MIR0917-351R. PMC6157004 29469956

[33] D. N. Clark , L. R. Begg , and A. J. Filiano , “Unique Aspects of IFN‐γ/STAT1 Signaling in Neurons,” Immunological Reviews 311, no. 1 (2022): 187–204, 10.1111/imr.13092. 35656941 PMC10120860

[34] H. Ding , G. Wang , Z. Yu , H. Sun , and L. Wang , “Role of Interferon‐Gamma (IFN‐γ) and IFN‐γ Receptor 1/2 (IFNγR1/2) in Regulation of Immunity, Infection, and Cancer Development: IFN‐γ‐Dependent or Independent Pathway,” Biomedicine & Pharmacotherapy = Biomedecine & Pharmacotherapie 155 (2022): 113683, 10.1016/j.biopha.2022.113683. 36095965

[35] M. Singh , M. Thakur , M. Mishra , et al., “Gene Regulation of Intracellular Adhesion Molecule‐1 (ICAM‐1): A Molecule With Multiple Functions,” Immunology Letters 240 (2021): 123–136, 10.1016/j.imlet.2021.10.007. 34715236

[36] A. Gérard , A. P. Cope , C. Kemper , R. Alon , and R. Köchl , “LFA‐1 in T Cell Priming, Differentiation, and Effector Functions,” Trends in Immunology 42, no. 8 (2021): 706–722, 10.1016/j.it.2021.06.004. 34266767 PMC10734378

[37] T. M. Bui , H. L. Wiesolek , and R. Sumagin , “ICAM‐1: A Master Regulator of Cellular Responses in Inflammation, Injury Resolution, and Tumorigenesis,” Journal of Leukocyte Biology 108, no. 3 (2020): 787–799, 10.1002/JLB.2MR0220-549R. 32182390 PMC7977775

[38] S. Ferrini , S. Sforzini , A. Cambiaggi , et al., “The LFA‐1/ICAM Cell Adhesion Pathway Is Involved in Tumor‐Cell Lysis Mediated by Bispecific Monoclonal‐Antibody‐Targeted T Lymphocytes,” International Journal of Cancer 56, no. 6 (1994): 846–852. 7907079 10.1002/ijc.2910560616

[39] M. Haustein , R. Ramer , M. Linnebacher , K. Manda , and B. Hinz , “Cannabinoids Increase Lung Cancer Cell Lysis by Lymphokine‐Activated Killer Cells via Upregulation of ICAM‐1,” Biochemical Pharmacology 92, no. 2 (2014): 312–325, 10.1016/j.bcp.2014.07.014. 25069049

[40] D. Kaiserlian , D. Rigal , J. Abello , and J. P. Revillard , “Expression, Function and Regulation of the Intercellular Adhesion Molecule‐1 (ICAM‐1) on Human Intestinal Epithelial Cell Lines,” European Journal of Immunology 21, no. 10 (1991): 2415–2421. 1680698 10.1002/eji.1830211018

[41] W. Dippold , B. Wittig , W. Schwaeble , W. Mayet , and K. H. Meyer zum Buschenfelde , “Expression of Intercellular Adhesion Molecule 1 (ICAM‐1, CD54) in Colonic Epithelial Cells,” Gut 34, no. 11 (1993): 1593–1597, 10.1136/gut.34.11.1593. 7902311 PMC1374428

[42] Y. Maruo , A. Gochi , A. Kaihara , et al., “ICAM‐1 Expression and the Soluble ICAM‐1 Level for Evaluating the Metastatic Potential of Gastric Cancer,” International Journal of Cancer 100, no. 4 (2002): 486–490. 12115535 10.1002/ijc.10514

[43] S. Wimmenauer , H. Keller , K. D. Rückauer , et al., “Expression of CD44, ICAM‐1 and N‐CAM in Colorectal Cancer. Correlation With the Tumor Stage and the Phenotypical Characteristics of Tumor‐Infiltrating Lymphocytes,” Anticancer Research 17, no. 4A (1997): 2395–2400. 9252653

[44] K. Maeda , S.‐M. Kang , T. Sawada , et al., “Expression of Intercellular Adhesion Molecule‐1 and Prognosis in Colorectal Cancer,” Oncology Reports 9, no. 3 (2002): 511–514. 11956618

[45] Y.‐C. Chen , E. Giovannucci , P. Kraft , R. Lazarus , and D. J. Hunter , “Association Between Toll‐Like Receptor Gene Cluster (TLR6, TLR1, and TLR10) and Prostate Cancer,” Cancer Epidemiology, Biomarkers & Prevention 16, no. 10 (2007): 1982–1989. 10.1158/1055-9965.EPI-07-0325 17932345

[46] M.‐F. Tsan , “Toll‐Like Receptors, Inflammation and Cancer,” Seminars in Cancer Biology 16, no. 1 (2006): 32–37, 10.1016/j.semcancer.2005.07.004. 16153858

[47] H. Sun , Y. Li , P. Zhang , et al., “Targeting Toll‐Like Receptor 7/8 for Immunotherapy: Recent Advances and Prospectives,” Biomarker Research 10, no. 1 (2022): 89, 10.1186/s40364-022-00436-7. 36476317 PMC9727882

[48] J. Cherfils‐Vicini , S. Platonova , M. Gillard , et al., “Triggering of TLR7 and TLR8 Expressed by Human Lung Cancer Cells Induces Cell Survival and Chemoresistance,” The Journal of Clinical Investigation 120, no. 4 (2010): 1285–1297, 10.1172/JCI36551. 20237413 PMC2846035

[49] H. Sumimoto , K. Tani , Y. Nakazaki , et al., “Superiority of Interleukin‐12‐Transduced Murine Lung Cancer Cells to GM‐CSF or B7‐1 (CD80) Transfectants for Therapeutic Antitumor Immunity in Syngeneic Immunocompetent Mice,” Cancer Gene Therapy 5, no. 1 (1998): 29–37. 9476964

[50] J. Liu and M. R. Johnston , “Animal Models for Studying Lung Cancer and Evaluating Novel Intervention Strategies,” Surgical Oncology 11, no. 4 (2002): 217–227. 12450558 10.1016/s0960-7404(02)00053-1

[51] S.‐Y. Sung , C.‐L. Hsieh , D. Wu , L. W. K. Chung , and P. A. S. Johnstone , “Tumor Microenvironment Promotes Cancer Progression, Metastasis, and Therapeutic Resistance,” Current Problems in Cancer 31, no. 2 (2007): 36–100, 10.1016/j.currproblcancer.2006.12.002. 17362788

[52] M. Hu and K. Polyak , “Microenvironmental Regulation of Cancer Development,” Current Opinion in Genetics & Development 18, no. 1 (2008): 27–34, 10.1016/j.gde.2007.12.006. 18282701 PMC2467152

[53] I. P. Witz , “The Tumor Microenvironment: The Making of a Paradigm,” Cancer Microenvironment 2 Suppl 1 (2009): 9–17, 10.1007/s12307-009-0025-8. PMC2756342 19701697

[54] F. Mbeunkui and D. J. Johann , “Cancer and the Tumor Microenvironment: A Review of an Essential Relationship,” Cancer Chemotherapy and Pharmacology 63, no. 4 (2009): 571–582, 10.1007/s00280-008-0881-9. 19083000 PMC2858592

[55] Y. Xiao and D. Yu , “Tumor Microenvironment as a Therapeutic Target in Cancer,” Pharmacology & Therapeutics 221 (2021): 107753, 10.1016/j.pharmthera.2020.107753. 33259885 PMC8084948

[56] H. Hu , Y. Chen , S. Tan , et al., “The Research Progress of Antiangiogenic Therapy, Immune Therapy and Tumor Microenvironment,” Frontiers in Immunology 13 (2022): 802846, 10.3389/fimmu.2022.802846. 35281003 PMC8905241

[57] N. Sengupta , T. S. MacFie , T. T. MacDonald , D. Pennington , and A. R. Silver , “Cancer Immunoediting and “Spontaneous” Tumor Regression,” Pathology, Research and Practice 206, no. 1 (2010): 1–8, 10.1016/j.prp.2009.10.001. 19945228

[58] Z. Li , F. Pradera , T. Kammertoens , B. Li , S. Liu , and Z. Qin , “Cross‐Talk Between T Cells and Innate Immune Cells Is Crucial for IFN‐Gamma‐Dependent Tumor Rejection,” Journal of Immunology (Baltimore, md. : 1950) 179, no. 3 (2007): 1568–1576. 17641023 10.4049/jimmunol.179.3.1568

[59] P. S. Goedegebuure , K. Y. Lee , Y. L. Matory , G. E. Peoples , I. Yoshino , and T. J. Eberlein , “Classification of CD4+ T Helper Cell Clones in Human Melanoma,” Cellular Immunology 156, no. 1 (1994): 170–179. 7911074 10.1006/cimm.1994.1162

[60] H. Sung , J. Ferlay , R. L. Siegel , et al., “Global Cancer Statistics 2020: GLOBOCAN Estimates of Incidence and Mortality Worldwide for 36 Cancers in 185 Countries,” CA: A Cancer Journal for Clinicians 71, no. 3 (2021): 209–249, 10.3322/caac.21660. 33538338

[61] A. A. Thai , B. J. Solomon , L. V. Sequist , J. F. Gainor , and R. S. Heist , “Lung Cancer,” Lancet 398, no. 10299 (2021): 535–554, 10.1016/S0140-6736(21)00312-3. 34273294

[62] C. M. Rudin , E. Brambilla , C. Faivre‐Finn , and J. Sage , “Small‐Cell Lung Cancer,” Nature Reviews. Disease Primers 7, no. 1 (2021): 3, 10.1038/s41572-020-00235-0. PMC8177722 33446664

[63] L. E. Quint , S. Tummala , L. J. Brisson , et al., “Distribution of Distant Metastases From Newly Diagnosed Non‐Small Cell Lung Cancer,” The Annals of Thoracic Surgery 62, no. 1 (1996): 246–250. 8678651 10.1016/0003-4975(96)00220-2

[64] H. H. Popper , “Progression and Metastasis of Lung Cancer,” Cancer Metastasis Reviews 35, no. 1 (2016): 75–91, 10.1007/s10555-016-9618-0. 27018053 PMC4821869

