# Supplementary material for: The Novel Fusion Protein Melittin‐MIL‐2 Exhibits Strong Antitumor Immune Effect in Lung Adenocarcinoma Cell A549
Source: Clin Respir J. 2024 Jul 14;18(7):e13805. doi: 10.1111/crj.13805 (PMC11246609; doi:10.1111/crj.13805)

细胞遗传质量鉴定检测

Cell Line Authentication Service

STR 基因型检测报告

送检单位：上海酶研生物科技有限公司

检品名称：细胞系

委托单位：上海翼和应用生物技术有限公司

报告日期：2016-09-22

上海酶研生

样 品 信 息

样品编号：

客户样本编号	公司编号
A549	20160910-01

样品数量：1

样品性状：细胞系

检测项目：STR

送检单位：酶研生物

检测方法：用 Axygen 的基因组抽提试剂盒提取 DNA，采用 20- STR 扩增方案扩增，在 ABI 3730XL 型遗传分析仪上对 STR 位点和性别基因 Amelogenin 进行检测。

检验结果：

(一)检验基本情况

预检样本	多等位基因	匹配细胞系	细胞库	匹配说明
A-549	无	A-549	DSMZ	完全匹配

- 多等位基因指三等位及以上基因现象。
- 本次检测各细胞分型结果良好。
- 该株细胞 DNA 分型在细胞系检索中找到完全匹配的细胞系，DSMZ 数据库显示细胞名为 **A-549 等**，细胞号对应 **107 等**。本次检测在该细胞系中没有发现多等位基因。
- **备注**：待测细胞系与收录于 ATCC, DSMZ, JCRB 和 RIKEN 数据库的细胞系 STR 数据进行比对，未收录于以上细胞库的细胞系将无法匹配。

（二）分型结果

表：预检样本的 STR 位点和 Amelogenin 位点的基因分型结果

Marker	样本				细胞库信息		
	Allele1	Allele2	Allele3	Allele4	Allele1	Allele2	Allele3
D5S818	11	11			11	11	
D13S317	11	11			11	11	
D7S820	8	11			8	11	
D16S539	11	12			11	12	
VWA	14	14			14	14	
TH01	8	9.3			8	9.3	
AMEL	X	Y			X	Y	
TPOX	8	11			8	11	
CSF1PO	10	12			10	12	
D12S391	18	18					
FGA	23	23					
D2S1338	24	24					
D21S11	29	29					
D18S51	14	17					
D8S1179	13	14					
D3S1358	16	16					
D6S1043	11	13					
PENTAE	7	11					

D19S433	13	13					
PENTAD	9	9					

其他说明：

(一)分型方案及位点分布：

附表：实验方案及位点

	方案 1	方案 2	方案 3	方案 4	方案 5	方案 6
1	TH01	AMEL	TPOX	D3S1358	PENTAE	D19S433
2	D12S391	D5S818	VWA	D13S317		PENTAD
3	D7S820	D2S1338	D8S1179	D6S1043		
4	CSF1PO	D21S11		D16S539		
5	FGA	D18S51				

(二)STR 数据库比对：

采用 DSMZ tools 进行细胞系比对，其中包含来自于 ATCC, DSMZ, JCRB 和 RIKEN 数据库的 2455 个细胞系 STR 数据。如果待检测细胞未收录于以上细胞库或这是自行建立的新细胞系将无法进行比对，用户需根据细胞分型结果自行与其他数据库进行比对。

Applied Biosystems
GeneMapper 4.0

Cell Line Authentication-2

Sample File	Sample Name	Panel	OS	SQ
65_A09_CellLineAuthentication09105-0919.FSA	035	STR Profile 1-dup		

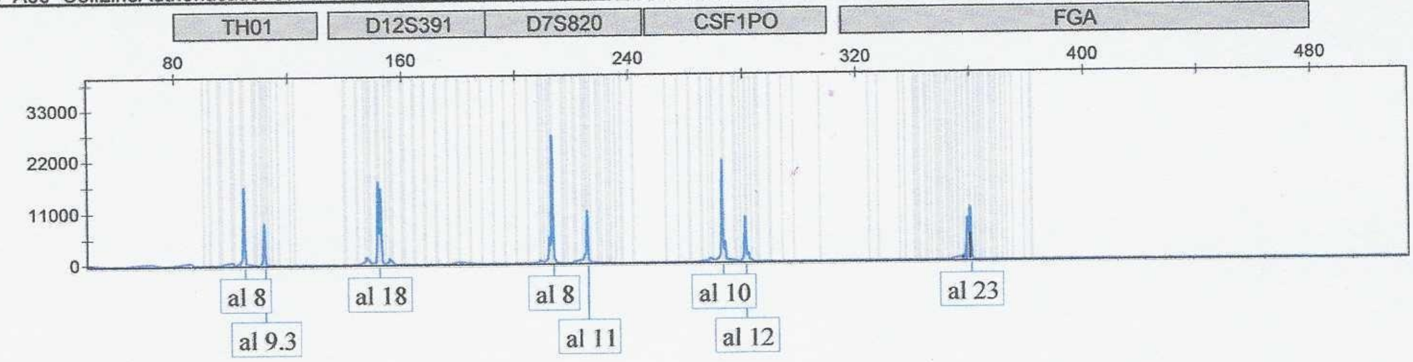

01_A01_CellLineAuthentication09122-0919.FSA	035	STR Profile 2-dup		

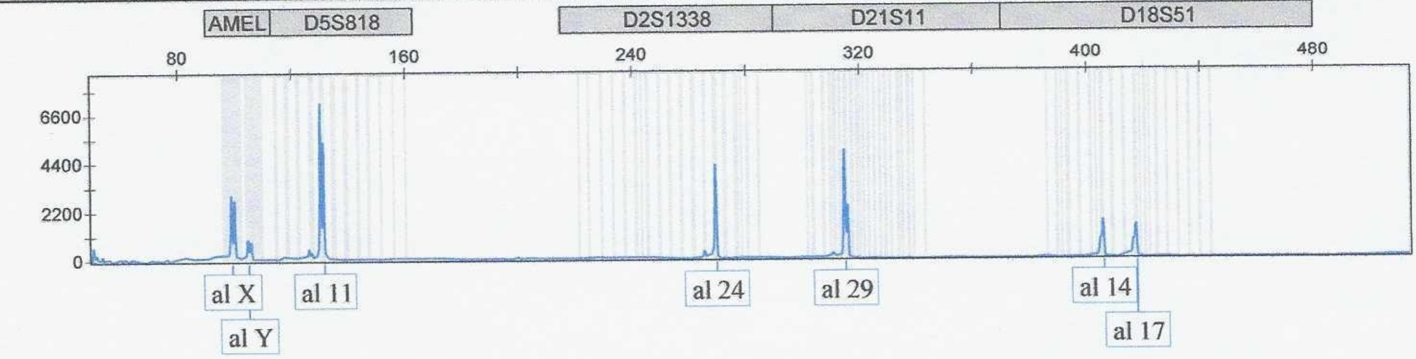

33_A05_CellLineAuthentication09122-0919.FSA	035	STR Profile 3-dup		

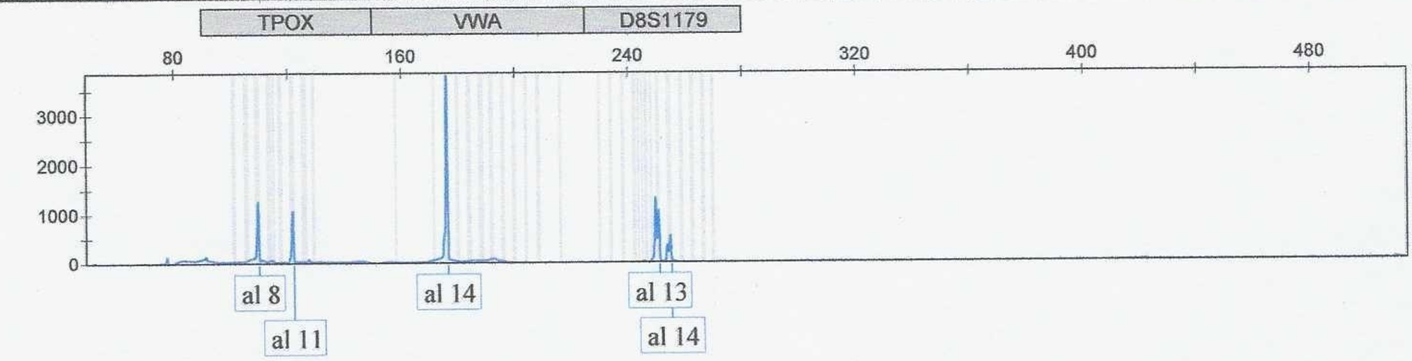

Applied Biosystems

apper 4.0

Cell Line Authentication-2

le File	Sample Name	Panel	OS	SQ
\09_CellLineAuthentication09122-0919.FSA	035	STR Profile 4-dup		

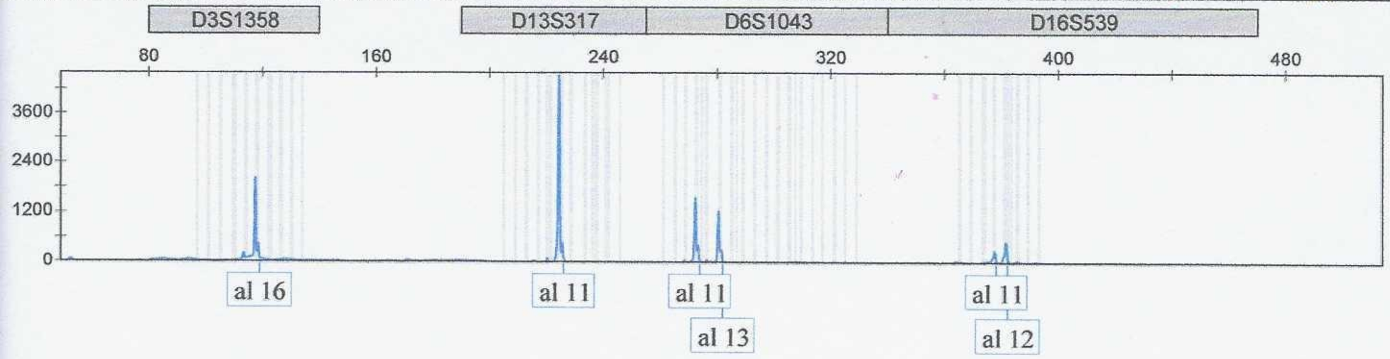

\01_CellLineAuthentication09125-0919.fsa	035	STR Profile 5-dup		

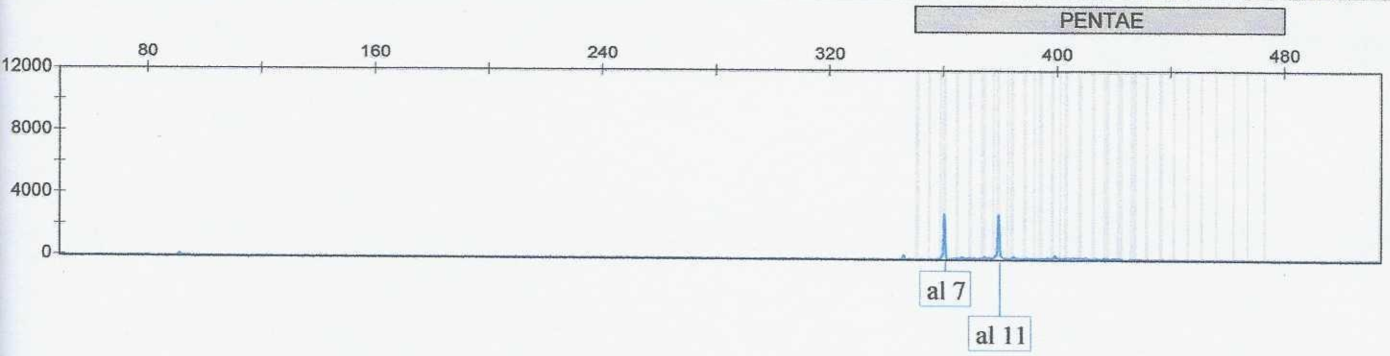

\05_CellLineAuthentication09125-0919.fsa	035	STR Profile 6-dup		

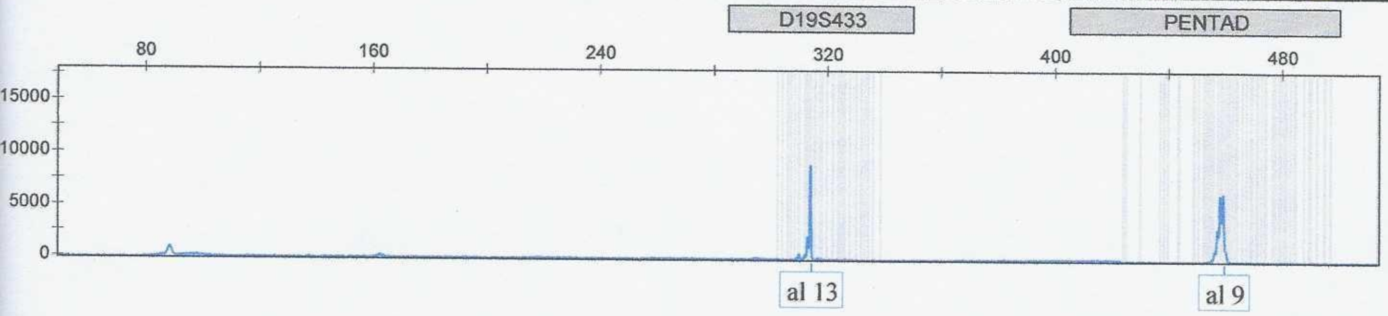

Supplement: Supplementary file 1 — Data S1 Supporting Information [file CRJ-18-e13805-s001.zip › supplementary materials/A549 STR report.pdf]
