# Supplementary figures and images for: The Novel Fusion Protein Melittin‐MIL‐2 Exhibits Strong Antitumor Immune Effect in Lung Adenocarcinoma Cell A549
Source: Clin Respir J. 2024 Jul 14;18(7):e13805. doi: 10.1111/crj.13805 (PMC11246609; doi:10.1111/crj.13805)

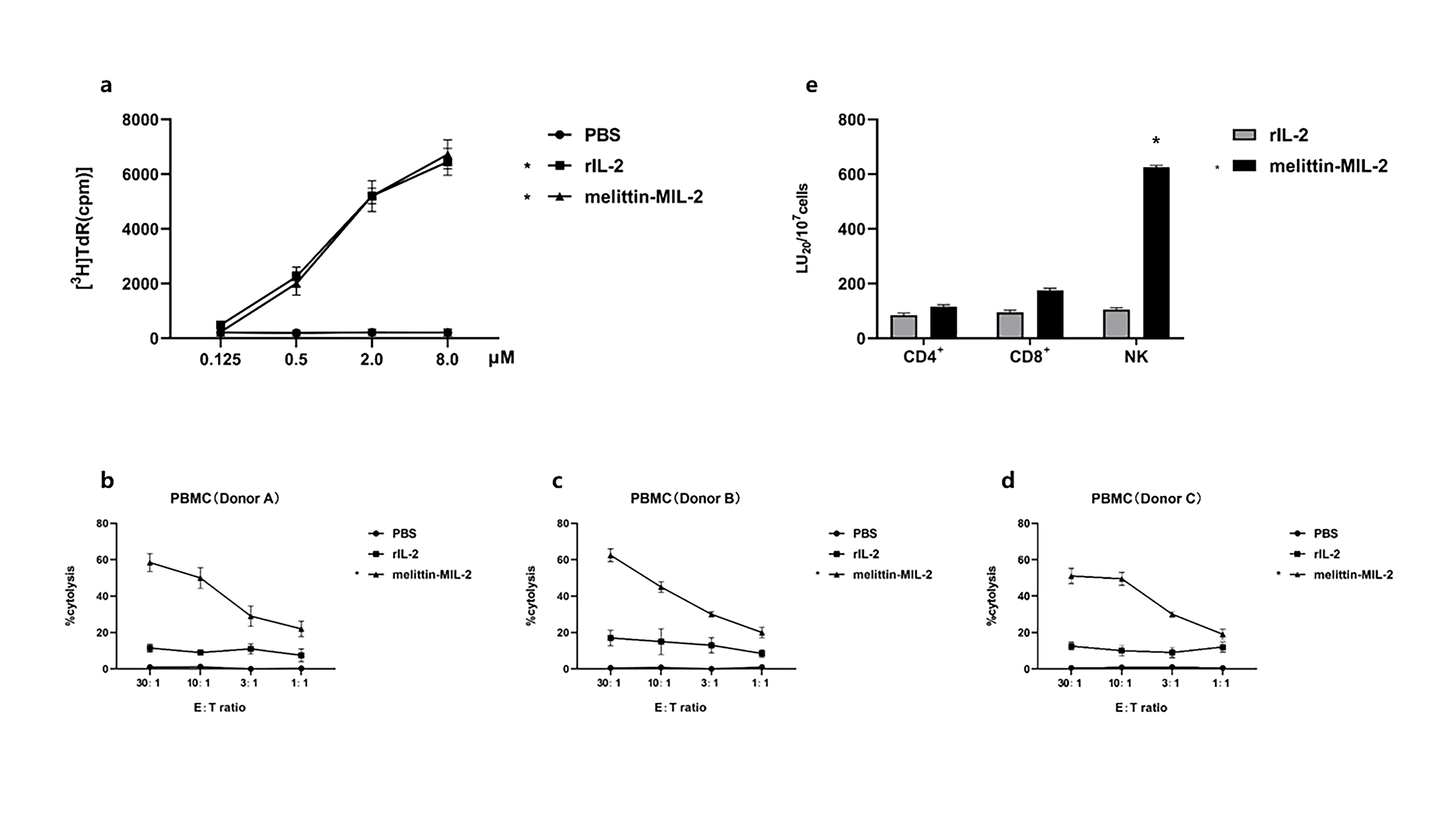

Supplement: Supplementary file 1 — Data S1 Supporting Information [file CRJ-18-e13805-s001.zip › supplementary materials/Figure S1.png]

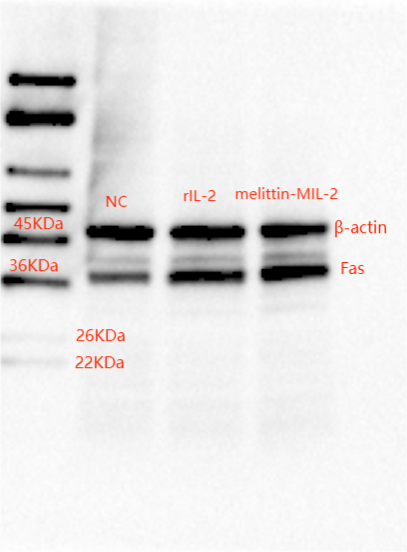

Supplement: Supplementary file 1 — Data S1 Supporting Information [file CRJ-18-e13805-s001.zip › supplementary materials/Western blot(ICAM-1 and Fas)/FAS-1.png]

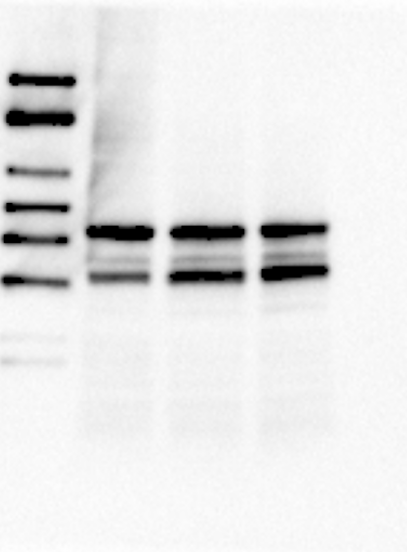

Supplement: Supplementary file 1 — Data S1 Supporting Information [file CRJ-18-e13805-s001.zip › supplementary materials/Western blot(ICAM-1 and Fas)/FAS-1.tif]

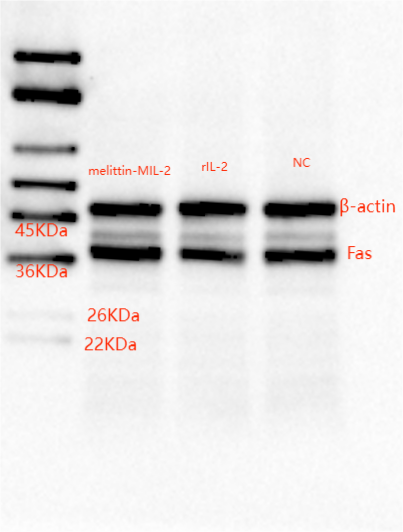

Supplement: Supplementary file 1 — Data S1 Supporting Information [file CRJ-18-e13805-s001.zip › supplementary materials/Western blot(ICAM-1 and Fas)/FAS-2.png]

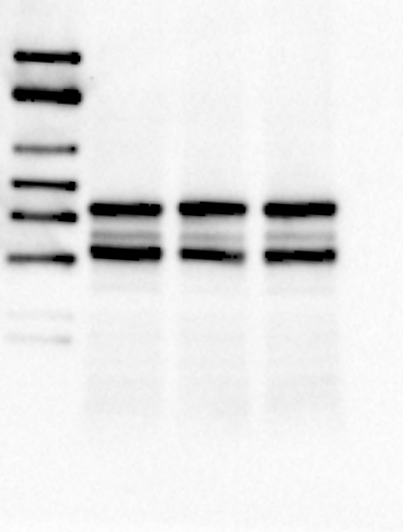

Supplement: Supplementary file 1 — Data S1 Supporting Information [file CRJ-18-e13805-s001.zip › supplementary materials/Western blot(ICAM-1 and Fas)/FAS-2.tif]

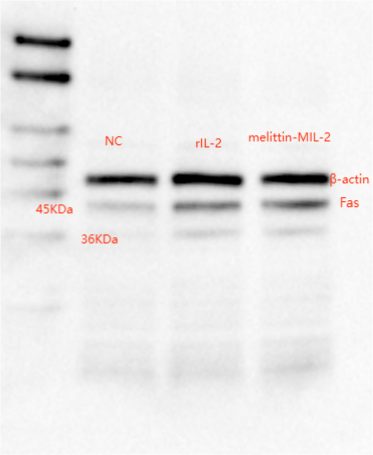

Supplement: Supplementary file 1 — Data S1 Supporting Information [file CRJ-18-e13805-s001.zip › supplementary materials/Western blot(ICAM-1 and Fas)/FAS-3.png]

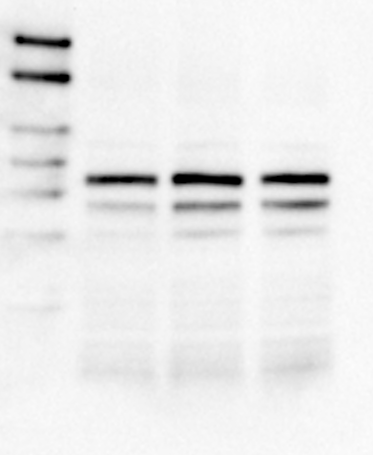

Supplement: Supplementary file 1 — Data S1 Supporting Information [file CRJ-18-e13805-s001.zip › supplementary materials/Western blot(ICAM-1 and Fas)/FAS-3.tif]

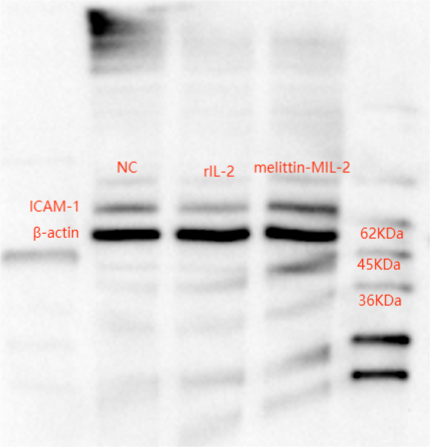

Supplement: Supplementary file 1 — Data S1 Supporting Information [file CRJ-18-e13805-s001.zip › supplementary materials/Western blot(ICAM-1 and Fas)/ICAM-1-1.png]

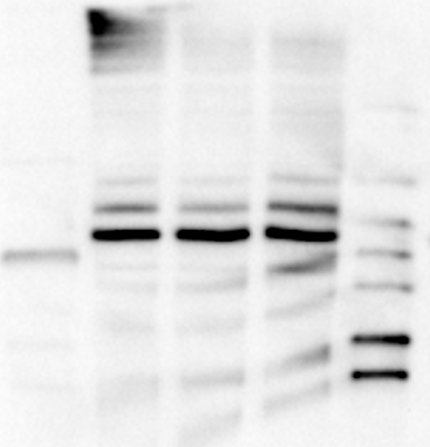

Supplement: Supplementary file 1 — Data S1 Supporting Information [file CRJ-18-e13805-s001.zip › supplementary materials/Western blot(ICAM-1 and Fas)/ICAM-1-1.tif]

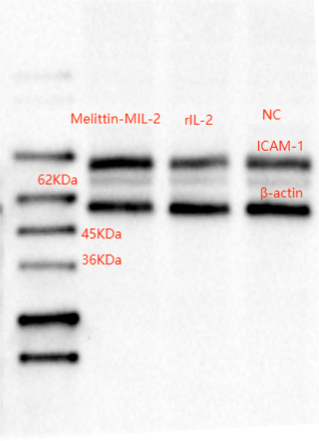

Supplement: Supplementary file 1 — Data S1 Supporting Information [file CRJ-18-e13805-s001.zip › supplementary materials/Western blot(ICAM-1 and Fas)/ICAM-1-2.png]

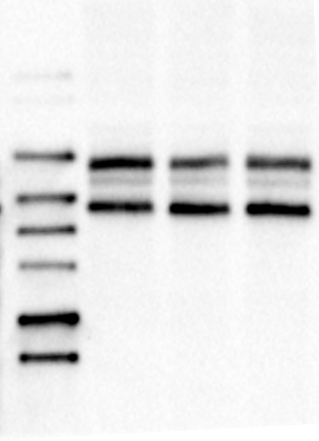

Supplement: Supplementary file 1 — Data S1 Supporting Information [file CRJ-18-e13805-s001.zip › supplementary materials/Western blot(ICAM-1 and Fas)/ICAM-1-2.tif]

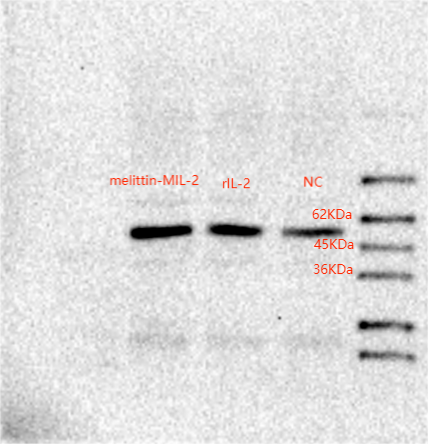

Supplement: Supplementary file 1 — Data S1 Supporting Information [file CRJ-18-e13805-s001.zip › supplementary materials/Western blot(ICAM-1 and Fas)/ICAM-1-3.png]

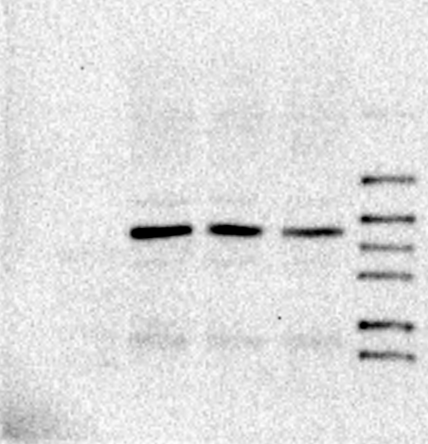

Supplement: Supplementary file 1 — Data S1 Supporting Information [file CRJ-18-e13805-s001.zip › supplementary materials/Western blot(ICAM-1 and Fas)/ICAM-1-3.tif]

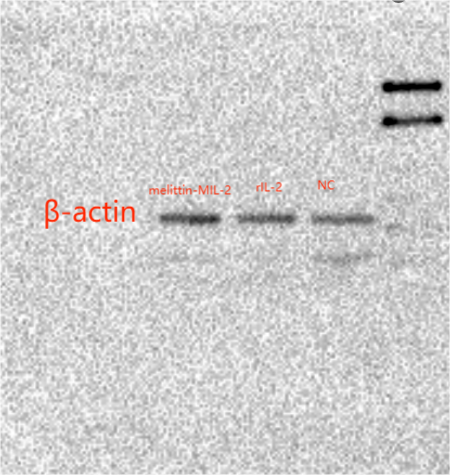

Supplement: Supplementary file 1 — Data S1 Supporting Information [file CRJ-18-e13805-s001.zip › supplementary materials/Western blot(ICAM-1 and Fas)/ICAM-3-β-actin.png]

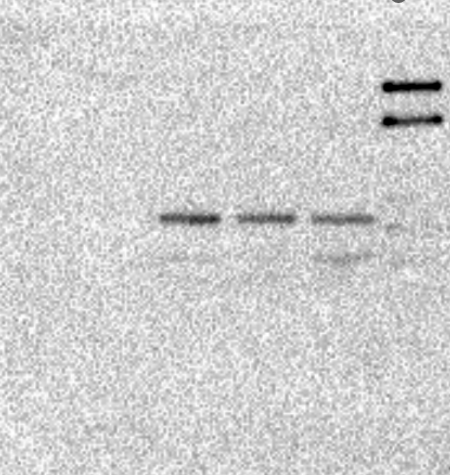

Supplement: Supplementary file 1 — Data S1 Supporting Information [file CRJ-18-e13805-s001.zip › supplementary materials/Western blot(ICAM-1 and Fas)/ICAM-3-β-actin.tif]
